# Wearable Gait Measurement System with an Instrumented Cane for Exoskeleton Control

**DOI:** 10.3390/s140101705

**Published:** 2014-01-17

**Authors:** Modar Hassan, Hideki Kadone, Kenji Suzuki, Yoshiyuki Sankai

**Affiliations:** 1 Graduate School of Systems and Information Engineering, University of Tsukuba, Tsukuba 305-8577, Japan; E-Mails: kenji@ieee.org (K.S.); sankai@golem.kz.tsukuba.ac.jp (Y.S.); 2 Center for Cybernics Research, University of Tsukuba, Tsukuba 305-8577, Japan; E-Mail: kadone@ccr.tsukuba.ac.jp; 3 Japan Science and Technology Agency, Saitama 332-0012, Japan

**Keywords:** wearable sensors, motion intention, exoskeleton robot, hemiplegia, cane

## Abstract

In this research we introduce a wearable sensory system for motion intention estimation and control of exoskeleton robot. The system comprises wearable inertial motion sensors and shoe-embedded force sensors. The system utilizes an instrumented cane as a part of the interface between the user and the robot. The cane reflects the motion of upper limbs, and is used in terms of human inter-limb synergies. The developed control system provides assisted motion in coherence with the motion of other unassisted limbs. The system utilizes the instrumented cane together with body worn sensors, and provides assistance for start, stop and continuous walking. We verified the function of the proposed method and the developed wearable system through gait trials on treadmill and on ground. The achievement contributes to finding an intuitive and feasible interface between human and robot through wearable gait sensors for practical use of assistive technology. It also contributes to the technology for cognitively assisted locomotion, which helps the locomotion of physically challenged people.

## 1. Introduction

Lower limbs exoskeleton robots offer major possibilities for support and rehabilitation of locomotion affected people [1–3]. Active exoskeleton robots can be used to augment human power [1], to support the locomotion of locomotion affected people [2,4,5], and to assist the process of rehabilitation as well [6–9]. Exoskeleton robots act directly on the human body, and are meant to assist human locomotion. Therefore, the design and control of these robots should be completely based on human characteristics, not only from ergonomics perspectives but also from motor control perspective as well. Also, compliance between the control system and different users is important. Thus, it is important to explore various human-machine interfaces and human motion intention estimation techniques, and to develop flexible control systems based on human motor control for the effective and proper use of exoskeleton robots.

**Figure 1.**
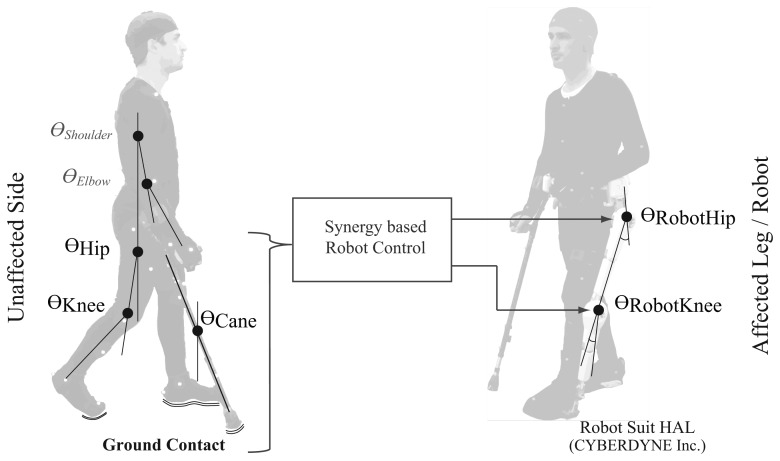
Illustration of the measured joint angles in the proposed system, and the concept of synergy based control.

**Figure 2.**
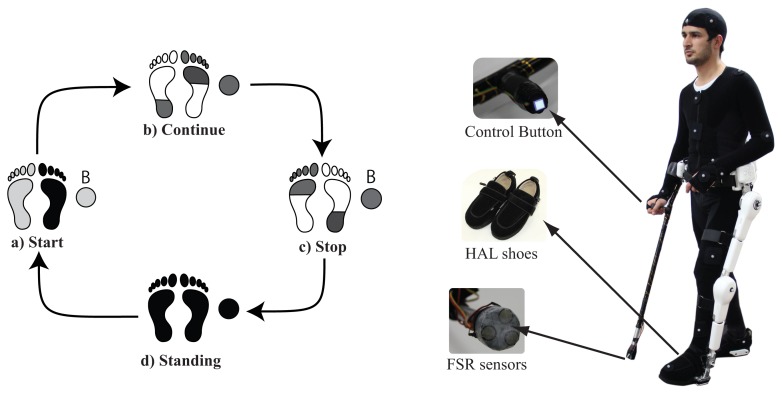
Start, walk and stop support based on ground contact patterns.

For assistance of locomotion affected people outside the laboratory environment, issues of human-machine interfacing, safety, wearability, and feasibility of the system should be considered. This paper addresses the development of a wearable gait measurement system with its underlying human gait characteristics and application to control of exoskeleton robot (Robot Suit HAL [1]). Robot Suit HAL is a wearable powered exoskeleton for support and rehabilitation of motor function in locomotion affected people. In recent studies the feasibility of rehabilitation training with HAL has been verified for stroke and spinal cord injury patients [8], and the locomotion improvement in chronic stroke patients after training with HAL was demonstrated as well [9]. The system in this work is designed for assistance of Hemiplegic persons with the single leg version of Robot Suit HAL. The single leg version is worn around the waist and on the affected leg, with straps around the thigh and shank segments to transfer the assist power to the leg. Power assist is provided through actuators at the hip and knee joints of the robot, while the ankle joint remains passive (Figures 1 and 2 show a person wearing the single leg version of Robot Suit HAL).

In recent years wearable systems for gait measurement and analysis gained significant improvements in feasibility and application [10–15]. These systems use inertial measurement sensors such as gyroscopes, accelerometers, and magnetometers for measuring the motion of limb segments and body parts. Also, force sensors embedded in shoe insole or underneath it are used for measurement of ground reaction forces and center of pressure in stance phases. Wearable sensors installed on the shoes [10–12] enable measurement and analysis of gait variables such as the stride length and width, single and double stance time, foot placement, and gait phases. Other wearable systems comprising inertial motion sensors fixed on lower limb segments [13,14] enable capturing the kinematics of lower limbs such as joints angles and limb orientation during ambulation.

The system we propose in this paper based on wearable technology is intended as an interface for real-time control of an exoskeleton robot by hemiplegic people. For the purpose of exoskeleton control application we consider inertial measurement sensors fixed on lower limb segments and force sensors embedded in the shoe insoles to capture lower limbs kinematics and ground contact information. Also, we consider using an instrumented cane as a mean for motion capture and motion intention estimation. While in other wearable systems the cane is not considered, we propose that in the case of hemiplegia the cane is incorporated in gait and, therefore, can provide valuable information for motion intention estimation and interfacing with an exoskeleton robot.

### 1.1. Related Work

Few interfaces have been developed for lower limbs exoskeleton robots, with the target pathology being hemiplegia or paraplegia. The bioelectrical signals are reliable information to estimate human motion intention [1]. However, in the case of neuronal injury/dysfunction such as Spinal Cord Injury (SCI) or stroke related paralysis, bioelectrical signals are different from that of healthy people or even not available. Therefore, reference trajectory for the assisted limb(s) needs to be computed, and the motion intention need to be estimated in different ways [2,4,5].

Kawamoto *et al.* [4] developed a control system for single leg version of Robot Suit HAL by using FRF (Floor Reaction Force) sensors to detect the gait phase shifting intended by the user. The readings from FRF sensors embedded in the shoe insole of the wearer were used to determine the current phase and phase shifting during gait, and the robot is then operated by assembling segments of reference trajectories extracted from healthy people to restitute the motion of the impaired limb. The reference trajectories are beforehand adjusted according to the user's physical conditions. More extended work has been realized for the case of paraplegia in [5]. In this work gyroscope, accelerometer and level sensors measure the tilting angle of the user's torso according to his anatomical lateral plane. And this information is also used for detecting the phase sequence intended by the wearer.

Krausser and Kazerooni [2] developed a Human Machine Interface for SCI people with an exoskeleton robot (eLEGS) and two crutches. The user convey his/her intention to the robot using the two crutches to perform Four-Point gait with assistance from the robot and the crutches. The sensor suit comprises load measurement mechanism on the crutches, inertial sensors on the arms, force sensors in the shoe insole, and angle sensors on the robot's actuators. The robot uses hip and knee angle measurements, foot pressure, arm angle, and crutch load to determine the current state and state transition in a state machine controller customized for Four-Point gait.

### 1.2. Proposal

#### Human Locomotion Synergies

The methods mentioned previously do not consider human inter-limb synergies in gait. Human gait is not only a function of the lower limbs, but also a coordination between upper and lower limbs as well [16–20], adding to balance and cognitive functions. Research on human locomotion have shown evidence for the existence of a task-dependent neuronal coupling of upper and lower limbs [21,22]. Also, research on inter-limb coordination after stroke [23] indicated that stroke patients in the acute stage have close to normal synergies in the unaffected side, and that synergies in the chronic stage depend on the level of recovery. It was also demonstrated that high functioning stroke patients preserve the ability to coordinate arm and leg movements during walking [24].

#### Proposed Approach

In this work we propose a system for control of exoskeleton robot by fusing sensory information from upper and lower limbs. We developed a wearable gait measurement system based on inertial measurement sensors and force sensors, and we fuse the sensory information for control of single leg version of Robot Suit HAL in real time. The system is targeted at persons with hemiplegia. In case of Hemiplegia, the person usually uses a cane in the unaffected arm (contralateral to the affected leg) to support body weight and balance [25,26]. Therefore, we propose to utilize an instrumented cane ,forearm-type crutch, as a part of the interface with the robot. We equip the cane with motion and force sensors to capture its motion, while it is still supportive for the user's balance and somatosesnory as a traditional walking aid.

#### Instrumented Cane

The cane as a walking aid does not only provide biomechanical support but also an augmentation to somatosensory, and therefore leads to enhanced posture control. John J Jeka [27] showed in a series of studies that “sensory input to the hand and arm through contact cues at the fingertip or through a cane can reduce postural sway in individuals who have no impairments and in persons without a functioning vestibular system, even when contact force levels are inadequate to provide physical support of the body”. Jeka's studies [27] were in quiet stance case. However, other studies showed similar benefits during ambulation. Rumpa *et al.* [28] showed that touch cue through the cane at weight acceptance of the paretic leg provides mediolateral pelvic stability for stroke persons.

The system devised by Krausser and Kazerooni [2] utilized two canes for motion intention estimation. The HMI they developed utilizes the ground contact of the cane and feet to allow four-point gait for paraplegic persons with an exoskeleton robot. Jang *et al.* [29] also explored walking intention estimation with a cane, but rather through motion sensors fixed on the hands (glove module) and contact force between the palm of the hand and the cane's handle. The mentioned examples utilize sensory information through the cane only for estimating the stepping intention. Thus, control of the exoskeleton is step-wise and segmented according to Three-Point or Four-Point gait patterns, considering the case of paraplegia. In the case of hemiplegia, on the other hand, the person has a nearly unaffected side on which he/she uses the cane. Therefore, we consider that the cane in this case could be used in a continuous manner, and could also accommodate the inter-limb synergies as well.

## 2. Methodology

### 2.1. Synergy Analysis

**Figure 3.**
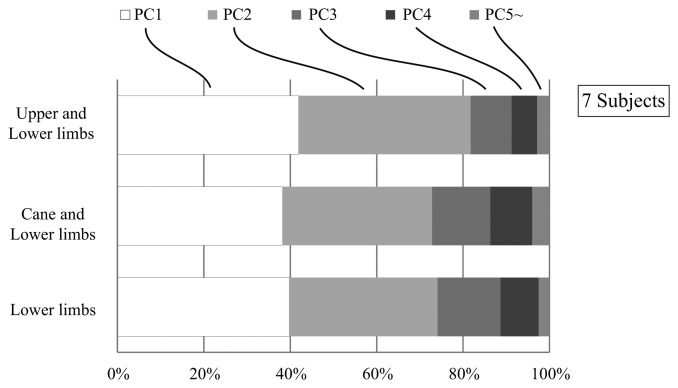
Group mean ratios of the first 4 principal components to the overall data for three sets of variables: (**i**) upper and lower limbs; (**ii**) cane and lower limbs; (**iii**) lower limbs.

In the proposed system we aim to use the cane to capture the unaffected arm motion, and to utilize it in the human machine interface with the robot based on its coordination with the lower limbs. Therefore, we first conducted an investigation to verify that the cane is incorporated in the joint coordination of upper and lower limbs [30]. In our investigation we asked seven healthy subjects to walk on a treadmill with/without a cane, and captured their kinematics with a 3D-Motion Capture System (see [30] for details). The joint angles and angular velocities of the shoulder, elbow, hip and knee joints for the right and left side limbs, as well as the tilting angle and angular velocity of the cane were computed in the sagittal plane (Figure 1). Three cases were inspected: (i) Joint coupling of the lower limbs; (ii) Joint coupling of the upper and lower limbs; (iii) Coupling of the cane and the lower limbs. We extracted and compared the synergies among the three cases by means of Principal Component Analysis (PCA). The results showed that for each of the three cases the first four synergies (represented by principal components) accounted for about 95% of the data variation (Figure 3). This result indicate that the cane motion falls into the synergies of upper and lower limbs in gait, and thus could be used in a synergy based control approach.

### 2.2. Motion Intention Estimation

In this work we consider a motion intention estimation method based on synergies of human locomotion. Vallery *et al.* [31,32] suggested a method called Complementary Limb Motion Estimation (CLME). In this method it is possible to compute the reference trajectory for affected limb(s) in real-time from the motion of other healthy (unaffected) limbs and the inter-joint coupling of healthy gait. In our investigation [30] we found that the cane is incorporated into the inter-joint synergies of gait. Therefore, we use the motion of the cane and the unaffected leg (considering the case of hemiplegia) together with the averaged synergies of walking with cane to estimate the motion of the affected leg. In this manner, the assisted motion will be automatically coordinated with the motion of the healthy leg and the cane (capturing the arm motion).

### 2.3. Start and Stop

Motion intention estimation based on CLME [31,32] generates the reference trajectory for the intended limb(s) based on synergies extracted from continuous walking. However, start and stop motions have different synergies from those of continuous walking. Therefore, it is necessary to provide support for start and stop motions separately, and to switch between start, continuous walking, and stop motions accordingly. Although some researches have shown possible the estimation of gait initiation before heel-off and toe-off [33]. Such studies are based solely on healthy patterns of gait, without consideration of disturbed patterns after pathology. Therefore we decided to build on a more feasible approach for estimation of start and stop intention that depends on the user actively conveying his/her intention. We provide a button on the handle of the cane (Figure 2), close to where the thumb would usually rest, that should be pushed before starting and stopping. Provided that the button is pushed, the system monitors the ground contact pattern on both feet using force sensors embedded in the shoes of Robot Suit HAL, and the cane's ground contact using FSR sensors on the tip of the cane. Start and stop motions are based on segments of trajectories extracted from walking with cane of healthy subjects. The control system switches between assistance of starting, continuous walking, and stopping according to the current gait status, button status, and ground contact patterns (Figure 2). This system will be explained in some more detail in a later section.

## 3. System Overview

### 3.1. Wearable System

The motion capture system is currently the most accurate mean for acquisition of human motion. However, systems based on inertial sensors for measurement and analysis of human motion (specially gait) have been steadily improving [13,14,34]. Using Inertial Measurement Units (IMUs) it is possible to capture the body motion by placing an IMU on each segment and fusing their information.

**Figure 4.**
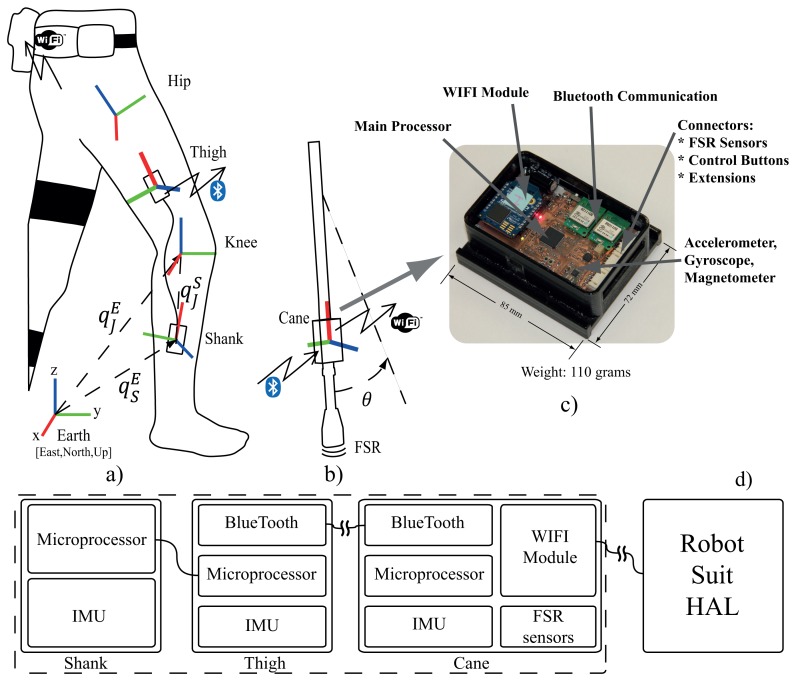
Wearable system configuration and frame calibration.

We developed a wearable gait measurement system based on inertial sensors, force sensors and embedded microprocessors to control exoskeleton robot. The system consists of three IMU modules: two modules fitted on the thigh and shank of the unaffected leg to acquire its motion (Figure 4a), and a main unit fixed on the cane (Figure 4b). Modules on the thigh and the shank acquire the motion (angle and angular velocity) of the hip and knee joints of the unaffected leg. The shank module is connected to the thigh module with wired serial communication, while the thigh module streams motion data from both thigh and shank modules to the main unit on the cane (Figure 4c,d). The module on the cane is the main unit (Figure 4c,d). It receives motion data via bluetooth from the thigh module, acquires the cane's motion (angle and angular velocity) from its own IMU, acquire the ground contact information from force sensors in the shoes of the robot through wireless communication , acquire the cane's ground contact information from FSR sensors, compute the control commands for the robot according to the current status, and stream those commands to the robot via WIFI communication. The force sensors embedded in the shoes consist of floor reaction force sensors under the heel and forefoot for each foot. The sensors provide continuous measurement of the floor reaction forces, and are used together with the FSR sensors on the tip of the cane to monitor the ground contact patterns for start-walk-stop support as well as for modification of control parameters in stance and swing phases (Figure 10).

### 3.2. System Calibration

The sensor fusion algorithm for IMU takes readings from 3-axis Gyroscope, 3-axis Accelerometer and 3-axis Magnetometer, and outputs the coordinates of sensor frame relative to reference frame (earth frame) in quaternion form. Performance of the algorithm is described in [35], accuracy; <0.8° static RMS error, <1.7° dynamic RMS error. In order to find the joint coordinates from the sensor coordinates a transformation is needed from the sensor frame to the joint frame. For performing this transformation we followed a procedure similar to that in recent methods [14,34]. The transformation from sensor frame to joint frame is given by Equation (6)

(6) $$q_{J}^{E}=q_{S}^{E}⊗q_{J}^{S}$$

The quaternions $q_{J}^{E}$, $q_{S}^{E}$ and $q_{J}^{S}$ represent the orientation of joint frame relative to earth frame, sensor frame relative to earth frame, and joint frame relative to sensor frame, respectively. And operator ⨂ is the quaternion multiplication. Therefore, to transform the sensor frame to joint frame we need to find the orientation of joint frame relative to sensor frame $q_{J}^{S}$. To do this we assume an initial position where the joint frame is known relative to earth frame. In our system we consider the initial position as quiet standing with the leg fully extended (leg completely vertical) and the person is roughly facing north. In this pose we assume that the joint frame for both hip and knee joints is identical to earth frame. From this position we can extract the quaternion of joint frame relative to sensor frame as in Equation (7)

(7) $$q_{J}^{S}={\left(q_{S}^{E}\right)}^{-1}⊗q_{J}^{E}$$

After calculating $q_{J}^{S}$ from the initial position we can use it to find the joint coordinates from the sensors coordinates assuming that the sensor mounting on the limb segment will not change while walking (sensor is attached firmly on the limb segment). We find the knee joint coordinates from the sensor fixed on the shank, and the hip joint coordinates from the sensor fixed on the thigh. Then we extract the joint angles in the sagittal plane since only motion in the sagittal plane is required in our system (the robot only provides assistance in the sagittal plane).

For the cane module this procedure was not required since the module is permanently fixed to the cane and well aligned to its axis. Therefore, just extracting the angle in the sagittal plan from the sensor's frame is adequate to produce the required cane's tilting angle.

### 3.3. Robot Control

In our work we use the single leg version of Robot Suit HAL. The hybrid control algorithm of Robot Suit HAL [1] consists of a human voluntary control and an autonomous control. The wearer's voluntary muscle activity is obtained from the bioelectrical signals, detected at the surface of the muscles, and then the required assist torque of the actuators is computed from the estimated joint torque. An autonomous control is also implemented based on the pre-determined motion primitives, together with the voluntary control method. In this work we provide the control reference to the robot from the developed wearable measurement system, and the robot's embedded motor control algorithm handles the execution. This modular approach for robot control allows for stacking additional modules of control in the future, allowing the capacity for further considerations such as balance monitoring and head orientation.

**Figure 5.**
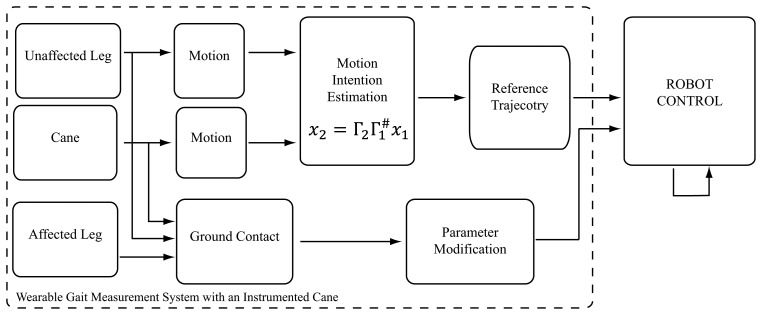
Schematic diagram of the control system in continuous gait.

Robot control with the developed wearable system will be explained here in detail. The system monitors the status of a start-stop button fitted on the handle of the cane and the ground contact patterns of the feet and the cane to detect start, walk, and stop conditions (Figure 2). We figured the start and stop conditions for this particular version considering the case of left side hemiplegia, where the user would be holding the cane with the right arm (unaffected side), and the robot would be fitted on the left leg (affected side). In this case we consider that the user would typically start with the left leg and the cane, since the right (unaffected) leg is more capable of supporting the body weight and balance requirements for starting. Accordingly, the start assist is triggered when the button is on, the right foot ground contact force is large, and the left foot and cane ground contact forces are small (Figure 2a). Transmission to the continuous walking mode is made at the next heel strike of the assisted leg, a state at which the unaffected leg is near to toe-off, and the cane is at contact with ground or close to it (Figure 2b). From this point assistance would be based on synergies based motion estimation from the cane and unaffected leg. Figure 5 illustrates the signal flow of the control system at this state. Motion of the affected leg's hip and knee joints are estimated from the motion of the cane and the motion of the unaffected leg's hip and knee joints (all motions are angle and angular velocities in the sagittal plane), as in Equation (8)

(8) $$x_{2}=\Gamma_{2}\Gamma_{1}^{\#}x_{1}$$

where *x*_2_ are the variables to be estimated: affected leg's hip and knee angles and angular velocities, *x*_1_ are the known variables: cane and unaffected leg's hip and knee angles and angular velocities, and $\Gamma_{2}\Gamma_{1}^{\#}$ is the rearranged matrix of the eigenvectors extracted from walking with cane trials of seven healthy subjects [30], and rearranged for estimation of *x*_2_ from *x*_1_ [31]. The estimated trajectories are streamed to the robot, and tracked with the actuators on the robot's hip and knee joints with PD controllers. The ground contact information from the robot's feet are used to modify control parameters in different conditions (Stance, Swing). To stop walking the user pushes the handle button again to release, then at the next heel contact of the unaffected leg (Figure 2c), toe-off of the affected leg, the stop motion would start, leading to quiet standing condition (Figure 2d). This pattern is also based on the stopping motion being supported by the unaffected leg, being more proper for hemiplegic persons.

## 4. Experimental Evaluation

We devised various experiments to verify the function and feasibility of the proposed approach and the developed system. We test the system here with healthy subjects to verify its function, and to inspect for needed adjustments before trials with persons with hemiplegia. We asked healthy subjects to walk on a treadmill with the proposed method being implemented with the wearable system and with a 3D-Motion Capture System (MOCAP). Experiments were done with a left leg version of Robot Suit HAL, with the cane being used in the right arm. In treadmill trials we only used the continuous walk support, to avoid any fall risks that could result from using the start and stop support on a treadmill. We evaluated the resulting gait variables for each case and compared the results among the two. Also, we asked one subject to test the wearable system on ground with start and stop support to evaluate the feasibility of those functions as well.

### 4.1. Subjects

We recruited four healthy male subjects for the experiments. All the subjects participated voluntarily, and neither had any history of locomotion deficits. Subjects had an average age of 26.5 ± 4.5 years, average weight of 62.6 ± 2 kg, and an average height of 172 ± 0.5 cm. All subjects signed a written informed consent, and all procedures were approved by the ethics committee of the University of Tsukuba.

### 4.2. Experimental Setup and Procedure

**Figure 6.**
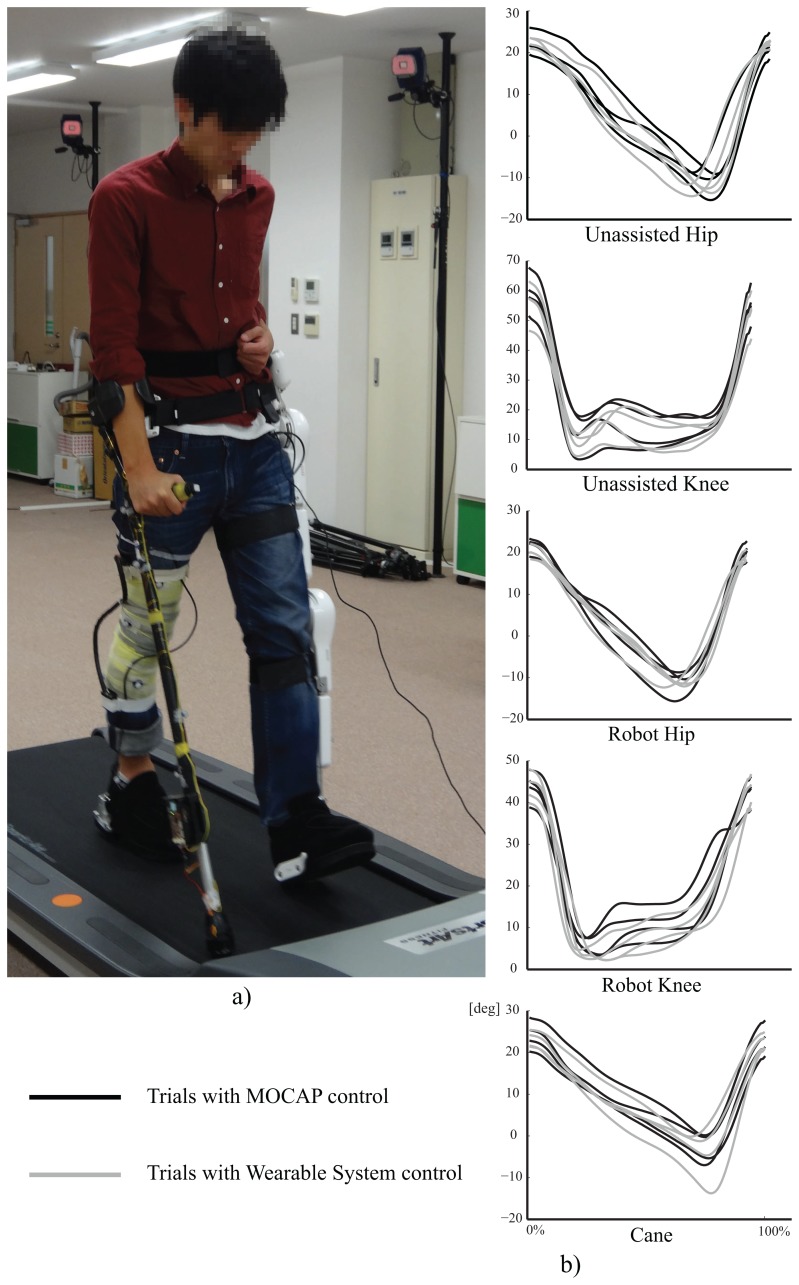
Experimental setup and average trajectories for all subjects.

The experimental setup for treadmill gait trials is shown in Figure 6a. The motion capture system was used for control and motion capture in the MOCAP trials, and only used for motion capture in trials with the wearable system. All the subjects used the cane in the right arm, and wore single leg version Robot Suit HAL on the left leg. All users used a magnetic safety key attached to the subject's waist and to the treadmill controller, the key will stop the treadmill automatically if a subject lags on the treadmill to avoid falling risk. Also, one of the experimenters constantly watched over the experiment with control over the exoskeleton robot so he could immediately switch the robot to free motion mode, such that it could be easily moved by the subject, in any cases of imbalance. The treadmill speed was set to 1.5 Km/h for all subjects. The length of the cane and the shank and thigh segments of HAL were adjusted to the individual comfort of each subject. Reflexive markers were fitted on the right leg thigh and shank segments, four on each, and the same on robot HAL. Markers were also fixed on the cane to be tracked by the motion capture system. All subjects were introduced to the structure and purpose of the system, and they were all encouraged to modify the cane's motion to reach a gait that is most convenient for them, and each walked at his preferred cadence. Each subject was allowed a test trial of about two minutes to get used to the system, and then we captured 2∼3 trials of walking with the system, each for about two minutes.

### 4.3. Treadmill Experiments with the Wearable System and with Motion Capture System

For walking trials on the treadmill with the wearable system, the shank and thigh IMU modules were fitted with rubber bands, and the cane's module was fitted on it using a custom made casing. The wearable units were calibrated as in Figure 4. The control system computed the reference trajectory for the left leg (hip and knee joints) using the the motion of the right leg (hip and knee joints) and the cane, together with averaged synergies of seven healthy subjects acquired from walking with cane trials [30]. The sensor fusion algorithm was run on each module at 128 Hz. The cane module receives motion data from the thigh module at 128 Hz, and is connected to the robot via wireless network for receiving the ground contact readings and streaming control commands at 32 Hz.

Also, we implemented the control system with a motion capture system MAC3D (Motion Analysis Inc.) for comparison with the wearable system. Using the motion capture system we captured the motion of the subject and the cane at 120 fps. From the frames of the motion capture system we computed the angles and angular velocities of the right leg hip and knee joints, and the cane's angle and angular velocity, all in the sagittal plane. The ground contact was obtained from FSR sensors installed at the tip of the cane and force sensors embedded in the shoes of HAL via wireless communication. The control commands were transmitted to HAL through wireless network every other frame of the motion capture system (60 Hz).

### 4.4. On-Ground Experiment with Start and Stop Functions

To verify the the function of the entire system with start and stop support, we asked one of the subjects to walk with the system on ground and recorded his motion and feet ground contact from robot HAL. The trajectories for start and stop support were extracted from three healthy subjects walking on ground with cane. The subject performed seven steps including the start and stop steps.

## 5. Results and Discussion

To verify and evaluate the function of the developed wearable system, and the proposed method in general, we extracted and compared the trajectories and step related gait variables from the walking trials. For each subject we extracted 10 consequent gait cycles from a trial of walking with the wearable system and 10 consequent gait cycles from a trial with the MOCAP system. Steps and gait cycles were marked by identifying heel-strikes of the right and left legs from the ground contact data. We selected the cycles as to avoid having more than 5 missing frames at any point. Then we interpolated any missing frames with cubic interpolation, and smoothed the trajectories with a two-pass, 4th order, zero phase shift, 6-Hz cut-off frequency butterworth filter [36].

Figure 6b shows the average trajectories for a complete gait cycle of measured joint angles. The trajectories shown are the averages of the extracted 10 gait cycles for each trial, with the gait cycle duration normalized for all trajectories to compare range differences and trends in those trajectories. The dark lines represent the trajectories for trials with MOCAP control, and the lighter lines represent the trajectories with wearable system control.

The trajectories show close to normal assisted motion trajectory on the robot's hip and knee joints, compared to that of the unassisted motion on the right leg's hip and knee trajectories. However, the range of motion in the robot's knee was smaller than that on the other side. This observation has several possible underlying causes. One is imperfections in the motion estimation algorithm which is based on linear approximation of the relationships between the variables (PCA) [30]. Another is the change in balance and anatomy resulting from wearing a robot on one side of the body. From the cane's trajectory we note some variation in range between the subjects, as we encouraged subjects to adjust the motion of the cane to reach more comfortable gait.

**Figure 7.**
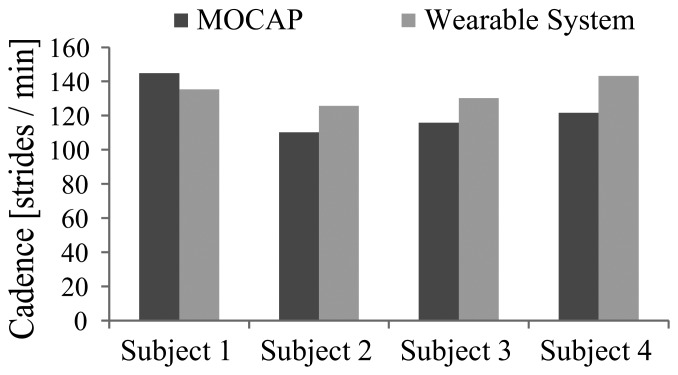
Cadence.

**Figure 8.**
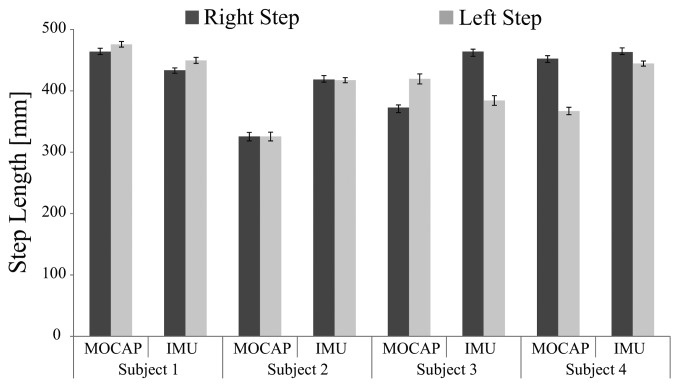
Right and Left Step Lengths.

**Figure 9.**
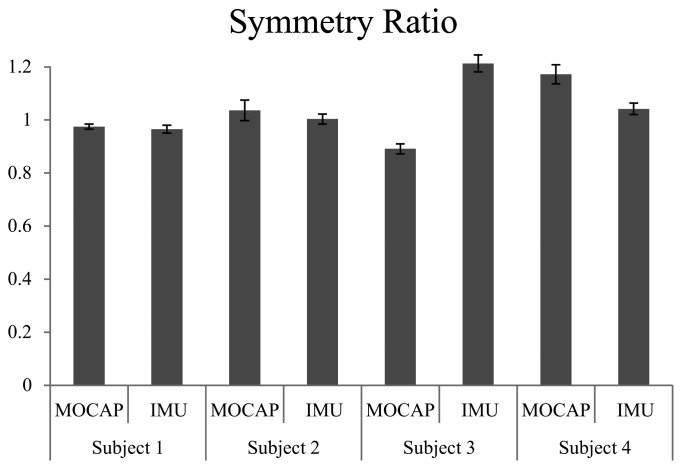
Symmetry Ratio (Right step/left step).

Figure 7 shows the cadence of each trial. Though subjects walked at the same speed on the treadmill, they had different body constitutions and walked at their own preferred cadence. Figure 8 shows the average step length on right and left sides, and Figure 9 shows the symmetry ratio of the trials. Subjects had slightly varying step length between the right and left sides. This is also seen in the symmetry ratio (considered here as the ratio of right step to left step). Figure 9 shows that subjects 1 and 2 achieved close to 1 (more symmetrical gait) ratios for both the wearable system and MOCAP trials, while subjects 3 and 4 had more varying symmetry ratios, closest to 1 is the wearable system trial of subject 4. We speculate that with the flexibility of the system, users would be able to habituate to it in rather a short while, and achieve more symmetrical gait patterns. We will focus on this issue in future studies to observe the changes in gait patterns with assisted motion over time.

**Figure 10.**
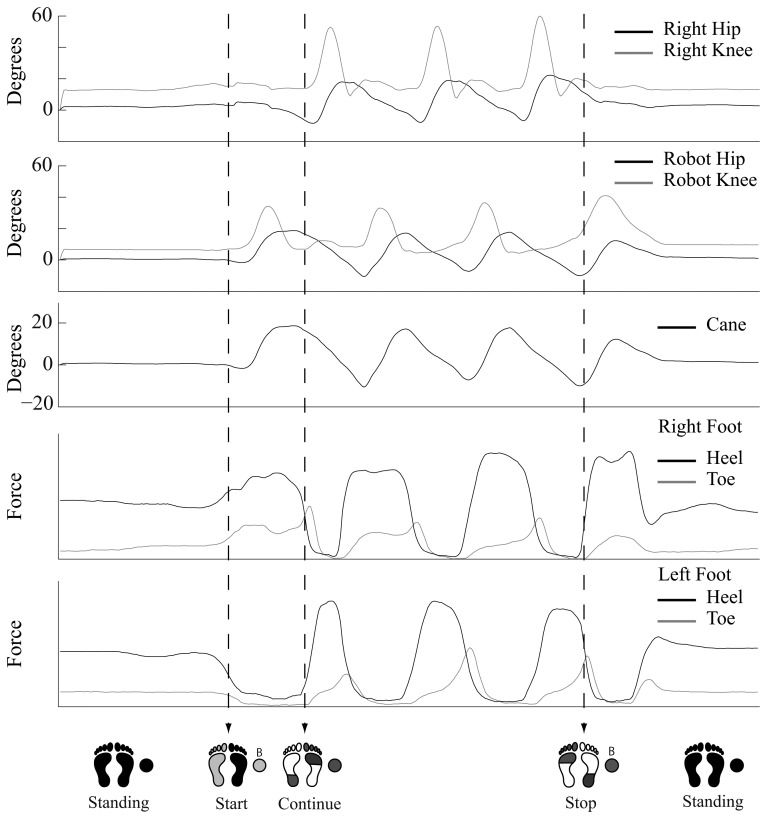
Start and Stop Support.

Figure 10 shows the joint trajectories and the ground contact pattern of the gait trial on ground with start and stop support. The trajectories shown from up down are the unaffected leg's hip and knee joints, the robot's hip and knee joints, the cane's tilting angle, the right foot sensors ground contact measurements, and the left foot sensors ground contact measurements. Underneath the trajectories are illustrations of the moments of transition, showing the successful transition from start to walk to stop motions. This experiment represents an aspect of basic locomotion assist such that for a hemiplegic person to start, walk, and stop with support from the exoskeleton robot. We consider that this scenario could also be implemented in robot assisted neuronal rehabilitation for a hemiplegic person. From the experiments we confirmed the feasibility of the developed wearable system for control of an exoskeleton robot with healthy subjects. All subjects used the system successfully and were able to use the wearable system to control the exoskeleton robot by using the instrumented cane as an interface with the robot for continuously and voluntarily guided support. However, we still need to run a pilot test with a hemiplegic person to verify the feasibility with a locomotion affected person. In the near future we look forward to having a pilot test with a hemiplegic person, and getting feedback on needed adjustments to the system. Then we may proceed to patient trials for assist and/or rehabilitation of hemiplegic people.

## 6. Conclusions

In this work we developed a wearable gait measurement system with an instrumented cane for control of an exoskeleton robot. The system utilizes the upper-lower limb coordination, which produces an assisted motion in harmony with unassisted limbs, and the body motion as a whole. We verified the function of the developed wearable system through trials of walking on treadmill, and comparison with similar trials by using motion capture system. The wearable system holds the advantages of being affordable and versatile for practical use.

By equipping the cane with motion and force sensors we were able to use it to capture the arm's motion, and to use it as an interface with the exoskeleton robot. We consider the instrumented cane also as a tool for gait measurement. It enables capture of the arm motion, and therefore the user intention. Measuring the arm motion directly could be prone to more cycle-to-cycle variation due to absence of the resetting effect of ground contact. The cane on the other hand extends the arm to the ground, which makes it more incorporated in gait, and also enables the benefits of light touch on balance and postural control.

Finding an intuitive and feasible interface between human and robot is essential for practical use of assistive technology. The wearable gait measurement system and robot control system suggested in this work represent a feasible approach for assistance and rehabilitation of locomotion affected people with an exoskeleton robot and an instrumented cane. With the proposed system it is possible to provide assistance in everyday life, and it is also possible to design new rehabilitation programs with consideration of upper-lower limbs coordination for physically challenged people.
